# Fracture diagnostics, unnecessary travel and treatment: a comparative study before and after the introduction of teleradiology in a remote general practice

**DOI:** 10.1186/s12875-015-0268-z

**Published:** 2015-05-06

**Authors:** Jac JWM Jacobs, Jan PAM Jacobs, Eric van Sonderen, Thys van der Molen, Robbert Sanderman

**Affiliations:** General Practice Ballum, 9162 ET Ballum Ameland, The Netherlands; Health Psychology Section, University Medical Centre Groningen, University of Groningen, 9712 CP Groningen, The Netherlands; Faculty of Economics and Business, University of Groningen, 9712 CP Groningen, The Netherlands; Department of Primary Care, University Medical Centre Groningen, University of Groningen, 9712 CP Groningen, The Netherlands; Department of Psychology, Health and Technology, University of Twente, 7522 NB Enschede, The Netherlands

**Keywords:** Teleradiology, Family practice, General practice, Diagnosis, Treatment, Trauma fractures

## Abstract

**Background:**

Teleradiology entails attainment of x-rays in one location, transfer over some distance and assessment at another location for diagnosis or consultation. This study documents fracture diagnostics, unnecessary trips to the hospital, treatment and number of x-rays for the years 2006 and 2009, before and after the introduction of teleradiology in a general practice on the island of Ameland in the north of the Netherlands.

**Methods:**

In a retrospective, descriptive, observational before and after study of the introduction of x-ray facilities in an island-based general practice, we compared the number of accurately diagnosed fractures, unnecessary trips, treatments and number of x-rays taken in 2006 when only a hospital x-ray facility was available 5 hours away with those in 2009 after an x-ray facility became available at a local general practice. All patients visiting a general practice on the island of Ameland in 2006 and 2009 with trauma *and* clinical suspicion of a fracture, dislocation or sprain were included in the study. The initial clinical diagnoses, including those based on the outcomes of x-rays, were compared for the two years and also whether the patients were treated at home or in hospital.

**Results:**

A total of 316 and 490 patients with trauma visited a general practice in 2006 and 2009, respectively. Of these patients, 66 and 116 were found to have fractures or dislocations in the two years, respectively. In 2006, 83 x-rays were ordered; in 2009, this was 284. In 2006, 9 fractures were missed; in 2009, this was only 2. In 2006, 15 patients with fractures or dislocations were treated at the general practice; in 2009, this had increased to 77.

**Conclusion:**

Since the introduction of teleradiology the number of missed fractures in patients visiting the general practice with trauma and the number of the unnecessary trips to a hospital are reduced. In addition more patients with fractures and dislocations can be treated in the general practice as opposed to the hospital.

## Background

Teleradiology is the electronic transmission of radiological images from one location to another for the purpose of interpretation and/or consultation. This technique has proliferated in many countries but not yet in the Netherlands [1]. In the Netherlands, all x-rays are obtained in hospitals or diagnostic centres and subsequently assessed by radiologists. In many other countries, x-rays are obtained in the general practices themselves and reviewed by the general practitioners (GPs). When judged necessary, a radiologist may sometimes be consulted with the use of teleradiology [2,3].

In the Netherlands, an average of 42–43 per 1000 patients experience new traumas and visit a general practice annually: 27 with strains on average; 13–14 with fractures on average; and 2 with dislocations on average [4]. For trauma patients with suspected fractures or dislocations, Dutch healthcare guidelines require x-ray confirmation of the fracture or dislocation in hospital, followed by either conservative or surgical treatment by a surgeon [5,6]. The GP in the Netherlands today normally refers the patient to the hospital for x-ray. Trauma patients with suspected strains, in contrast, are typically treated only on the basis of clinical signs by general practitioner.

In a relatively remote location, the island of Ameland in the north of the small country of the Netherlands, teleradiology was recently introduced. Prior to 2007, all patients with suspected fractures received plaster splints at the general practice for immobilization or when necessary following deformity correction, and were sent to the hospital for further x-ray examination (which is in keeping with the normal procedure in The Netherlands). These patients frequently returned with the same plaster splints following x-ray confirmation of the fracture or successful repositioning. In fact, at that time, such trauma patients often only travelled to the hospital to have the x-rays taken. Given that the hospital takes a ferry trip to be reached, the threshold for a referral to the hospital was (and is) very high. The physical examination at the general practice had to strongly suggest a fracture or dislocation for referral to the hospital; fractures of the phalanx (i.e., fingers or toes) or habitual shoulder dislocation were often treated in the general practice without x-ray back then.

Medical diagnosis always has the risk of missing something, on the one hand, versus unnecessary referral, on the other hand (i.e., patients travelling to hospital for nothing in the end). This dilemma and particularly the high threshold for ordering supplemental diagnostics in a rural location as Ameland was expected to disappear when a GP obtained access to an x-ray facility and introduced teleradiology to communicate with a hospital (i.e., radiologists and surgeons).

Telemedicine has received considerable attention in the research literature but teleradiology much less [3]. In the present study, it was therefore decided to investigate the following question: what is the influence of the introduction of an x-ray facility in a remote GP practice on accurately diagnosed fractures, hospital visits, number of x-rays and treatment. It was expected that the number of missed fractures and unnecessary hospital referrals (trips to the hospital) would decline with the introduction of teleradiology. We did not expect huge changes in treatment and number of x-rays, i.e. that clinical indications for x-rays would be unaffected.

## Methods

### Setting and preparation

Ameland is an island with 3500 inhabitants and 20 times as many tourists during the busy season (summer). Medical care is delivered at two general practices, which also in cases of emergencies serve the function of emergency room. The nearest hospital is in Dokkum on the mainland, with a travelling time of approximately five hours, including a ferry trip.

The teleradiology facility is installed in one of the two general practices but available for use with all patients — including those from the other GP on the island and tourists. When needed, the x-rays are taken by a trained radiographer working in the general practice and digitally transmitted to the hospital in Dokkum where the x-ray information is evaluated and interpreted by a trained radiologist. The radiologists are available during regular office hours and for emergency situations 24 hours a day, 7 days a week. In consultation with the surgeon in the hospital, it is decided whether the patient in question can be treated in the general practice under the supervision of a surgeon or should be treated in hospital. The radiographer is a full-time employee of the general practice and responsible only for the taking of x-rays and not for the interpretation of these.

The radiologist always responds digitally on the same day and, if necessary, directly by phone. The radiologist may sometimes give the radiographer special instructions for the x-rays by phone. The hospital’s x-ray protocol is followed. The radiographer receives ongoing feedback on the quality of the x-rays taken. And the radiographer receives annual training at the hospital.

The indications for an x-ray are twofold, namely: 1) trauma in the form of fractures or dislocations and 2) non-trauma requiring x-ray for monitoring or surgical purposes (e.g., x-ray in cases of hip degeneration, knee problems, and lung carcinoma).

In the present setting, the GP was trained as a radiation expert. Together with the Institute of Nuclear Services for Energy, Environment and Health in the Netherlands, the GP is also responsible for all radiation hygiene and safety within the general practice. The costs of the x-rays and the honorarium for the radiologist are covered by the patient’s insurance. The x-rays made in the general practice are stored together with any x-rays made at the hospital in the Picture Archiving and Communication System (PACS) of the hospital.

### Study design

In a retrospective, descriptive, observational before and after study, we compared the health outcomes for patients who visited the general practice with a recent trauma in 2006 — the year before the introduction of the teleradiology facility — and patients who visited the GP with a recent trauma in 2009 — the second year after the introduction of the facility and the most recent year for which data was available. Only traumas related to the musculoskeletal system (i.e., strains, fractures and dislocations) were investigated.

### Study population, data collection and material

Retrospective, all the patients who visited the GP in 2006 and 2009 with the above mentioned traumas were selected from the Promedico database by the GP himself. On the basis of their initial clinical signs, the patients were categorized into six groups: (1) clear deformity, (2) pain due to weight bearing or axial compression, (3) local pressure pain, (4) haematoma, (5) stiffness, (6a) no disorder or (6b) immobilized. Patients in group 1 definitely had a fracture or dislocation and needed treatment as soon as possible — preferably following x-ray confirmation of the condition. Patients in group 2 had suspected fractures which had not yet been confirmed but called for an x-ray. Patients in group 3 had strains but also the possibility of fracture(s) and were instructed to return for re-examination if still in doubt about the diagnosis after two days [4,5]. Patients in groups 4, 5 and 6a showed minimal trauma and no apparent fracture. The patients in group 6b had been immobilized (re-trauma), which precluded physical examination in the general practice.

Information was also gathered from the above mentioned database on the clinical diagnosis, whether an x-ray was obtained or not, undertaken treatment, location of treatment (hospital or general practice), the practice with which the patient was registered and the x-ray was ordered (GPs from both general practices on the island could order x-rays) and final diagnosis. A physician assistant contacted those patients for whom no final information on the medical outcome was available to obtain this information by telephone (i.e., both tourists and islanders who did not return to the practice following consultation for the relevant trauma were contacted to obtain required follow-up information). Table 1 lists the questionnaire.

**Table 1:** **Questionaire teleradiology**

1	When did you visit the general practice in 2006 or 2009?
2	What was the reason for the consultation?
3	What was the diagnosis?
4	Was the diagnosis correct?
5	How long did you have complaints?
6	Was an X-ray taken?
7	Question six if yes, when was that X-ray taken?
8	And what was the diagnosis?

Subsequently the GP anonymized the selected data (including the information gathered by the physician assistant) and a medical student imported these data into a registration system of the University Medical Centre Groningen. *International Classification of Primary Care* (ICPC) codes were assigned. The initial ICPC diagnoses (diagnosis at the moment of treatment) were then compared to the final ICPC diagnoses (diagnosis collected after a period by the physician assistants phone call or from the medical outcome).

### Ethics statement

Because the study is retrospective with data anonymized from patients records, it falls outside the Medical Research Involving Human Subjects Act (WMO) in the Netherlands and does not need to be approved by a medical ethics committee. We followed the Health Research Guidelines (Gedragscode Gezondheids Onderzoek 2004), which are based on the Medical Treatment Law (WGBO) and the Privacy Protection Law (Wbp). The use of anonymized data in medical research that cannot be traced back to individual patients is allowed. This study is based on anonymized medical records of the GP, which were completed by information obtained from patients after informed consent by a physician assistant.

## Results

In 2006 and 2009, respectively, 316 and 490 patients visited the general practice with recent traumas (see Table 2). From these 56 (2006) and 77 (2009) were contacted by phone; 4, 7 respectively could not be reached and one patient in 2009 refused to answer the questionnaire. Hence, our sample consists of 312 patients in 2006 and 482 patients in 2009.

**Table 2:** **Trauma patients visiting the general practice in 2006 and 2009**

	**2006**	**2009**
Number of trauma patients	316	490
Number of patients called by phone	60	85
Number of patients not reached/refused to answer	4	7/1
Study sample	312	482
X-rays	83 (26.6%)	281 (58.3%)
Hospital referrals	83 (26.6%)	39 (8.1%)
Unnecessary trips to the hospital	41 (13.1%)	2 (0.4%)
Fractures or dislocations	66 (21.2%)	116 (24.1%)
Fracture or dislocation treatment by the GP	15 (22.7%)^1^	77 (66.4%)^1^
Missed fractures	9 (13.6%)^1^	2 (1.9%)^1^

Note: ^1^Percentages of fractures.

In 2006, 83 patients (26.6%) were referred to hospital. For 41 of them (49.4%), this trip proved unnecessary; they did not have fractures and were treated further by the GP. In 2009, 39 patients (8.1%) were referred to hospital: 3 of these directly without x-ray in the general practice; 2 with a CT scan indication due to high-energy trauma; and 1 with a complicated tibia/fibula fracture. In retrospect, the two trips for the patients with the CT indications (0.4%) proved only precautionary. In 2006, a total of 83 x-rays were taken on 26.6% of the total number of patients visiting the general practice for recent trauma. In 2009, 281 x-rays were taken on 58% of the total number of trauma patients visiting the general practice.

In 2006, 66 (21%) of the patients had fractures or dislocations and 9 (13.6%) of these were missed. In 2009, 116 (24.1%) of the patients had fractures or dislocations and 2 (1.7%) of these were missed. The general practitioner treated 15 patients (22.7%) without x-ray confirmation in hospital in 2006 and 77 patients (66.4%) after x-ray confirmation in the general practice itself in 2009.

The majority of the fractures were radius/ulna, phalanx, metacarpal and tibia/fibula fractures (see Table 3). The 9 missed fractures in 2006 consisted of 3 radius/ulna, 4 tibia/fibula and 2 vertebral fractures. These fractures were much more severe than the 2 missed toe phalanx fractures in 2009.

**Table 3:** **Fractures and/or dislocations in 2006 and 2009**

**Year**	**2006**			**2009**		
	**Total (%)**	**Referred (%)**	**Missed (%)**	**Total (%)**	**Referred (%)**	**Missed (%)**
	**66**	**42 (63.6%)**	**9 (13.6%)**	**116**	**37 (31.9%)**	**2 (1.7%)**
Radius/ulna	13 (19.7%)	10 (76.9%)	3 (23%)	39 (36.1%)	14 (35.9%)	0
Tibia/fibula	10 (15.2%)	6 (60%)	4 (40%)	12 (11.1%)	3 (27.3%)	0
Metacarpal/phalanx	26 (39.4%)	13 (52%)	0	24 (22.2%)	5 (23.8%)	2 (8.3%)
Others	17 (25.8%)	12 (75%)	2 (12.5%)	33 (30.6%)	15 (35.7%)	0

The breakdown of the trauma patients with suspected fracture or dislocation on the basis of initial physical examination is as follows (see Table 4). In 2006, 23 trauma patients (8.5%) were identified as having deformities (group 1); in 2009, this was 37 (9.6%). In 2006, 20 of these patients were sent to the hospital for x-ray confirmation of the deformity after correction and immobilisation at the general practice; 3 of them had habitual shoulder dislocations and were treated by the GP without a visit to hospital. In 2009, 36 of the 37 trauma patients with suspected deformities (group 1) had an x-ray confirmation at the general practice before correction and/or immobilisation of the dislocation and x-ray checking again afterwards. In the end, 31 of these patients — including the patient mentioned above with the complicated tibia/fibula fracture — were sent to hospital for further treatment in 2009 and 6 were treated solely in the general practice.

**Table 4:** **Physical examination, diagnosis, and treatment in trauma patients in 2006 and 2009**

	**2006**						**2009**					
**Physical examination**	**1: Deformity**	**2: Axial compression pain**		**3: Local pressure pain**		**Total: 1, 2, 3 2006**	**1: Deformity**	**2: Axial compression pain**		**3: Local pressure pain**		**Total: ** **1, 2, 3 2009**
**1. Number of patients**	23	67		181		271	37	162		186		385
**2. Suspicion of fracture** ^**1**^		**Direct**	**After two days**	**Direct**	**After two days**			**Direct**	**After two days**	**Direct**	**After two days**	
**a. No**	0	9	0 + 4^2^	181	165 + 5^2^	165 + 9^2^	0	0	0	168	119 + 2^2^	119 + 2^2^
**b. Yes**	23	58	5	0	11	97	37^3^	162	0	16 + 2^4^	47	264
**3. Treatment by GP without x-ray** ** c. No fracture**	0	0	4	181	170	174	0	0	0	168	121	121
** b Fracture**	3^5^	12^6^	0	0	0	15^5,6^	0	0	0	0	0	0
**4. Result of x-ray (hospital in 2006 and general practice in 2009):** ** d. No fracture**	0	31	2	0	8	41	0	103	0	6 + 2^4^	47	158
** e. Fracture**	20	15	3	0	3	41	36 + 1^3^	59	0	10	0	106
**5. Treatment by GP after x-ray: f. No fracture**	0	31	2	0	8	41	0	103	0	6 + 2^4^	47	158
** g. Fracture**	0	0	0	0	0	0	6	53	0	10	0	69
**6. Treatment Hospital: fractures (only)**	20	15	3	0	3	41	31	6	0	0	0	37

Notes:

^1^Fractures = Fractures and/or dislocations.

^2^Missed fractures.

^3^One patient with a complicated tibia/fibular fracture was sent directly to the hospital without obtaining an x-ray at the general practice.

^4^Two patients with a high-energy trauma were sent directly to the hospital (CT-scans indicated).

^5^Three patients with habitual shoulder dislocation were treated by the GP without x-rays.

^6^Twelve patients with phalanx fractures were treated by the GP without x-rays.

The group of patients with axial compression pain (group 2) consisted of 67 (24.7%) patients in 2006 and 162 (42.1%) in 2009. Of these patients, 46 and all 162 had x-rays taken for 15 and 59 fractures, respectively. In 2006, 5 patients returned after two days for repeated x-ray and three of them were found to have fractures.

The group of patients with local pressure pain (group 3) consisted of 181 (66.8%) patients in 2006 and 186 (48.3%) in 2009. Of these patients, 16 of 2009 had an x-ray taken directly with 10 fractures and 11 and 47, respectively, had x-rays taken after two days. In 2006, 3 of these 11 patients were found to have fractures; in 2009, none of the 47 patients undergoing follow-up x-ray were found to have fractures. In 2009, 19 patients with a new trauma which occurred while in plaster immobilisation for a previous fracture (group 6b) had an immediate x-ray; 8 of them had a re-fracture and were further treated at the general practice. The group of patients with minor trauma (group 4, 5, 6a) consisted of 41 patients in 2006 and 97 in 2009. In 2006 one patient complained of stiffness and was immobilized transported to the general practice by an ambulance because of a high energy trauma. He was sent directly to the hospital with a cervical vertebra fracture suspicion where it was confirmed and treated.

**Figure 1:**
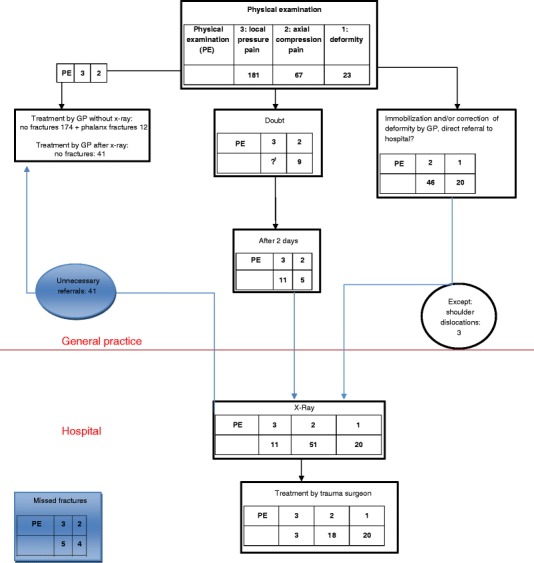
Flow chart for 2006.

**Figure 2:**
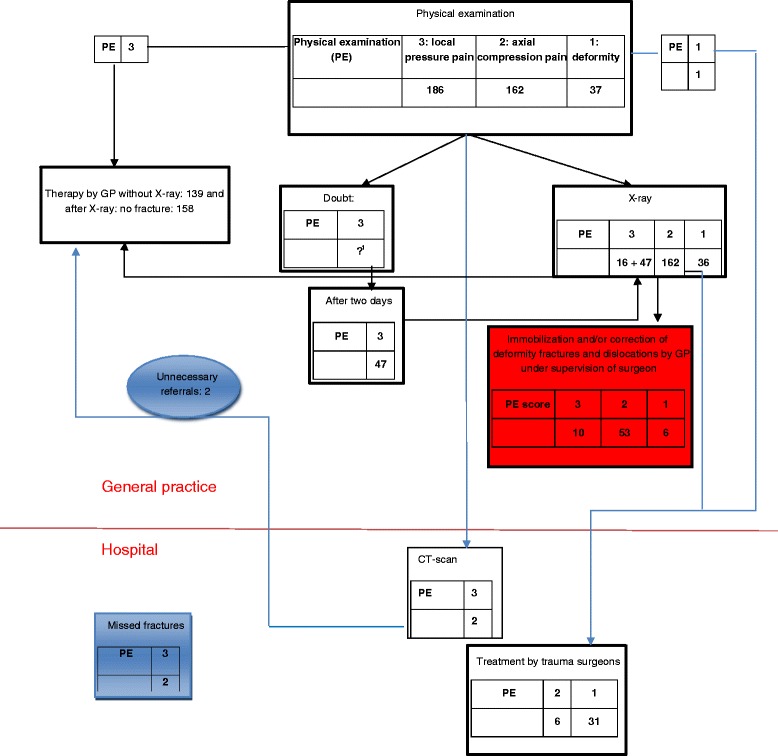
Flow chart for 2009. Note: 1. In principle no doubt. But if complaints remain after two days return to practice.

Figures 1 and 2 in the Appendix connect the results presented in Tables 2 and 4. Clear differences in the thresholds for x-ray (82 at hospital in 2006; 281 at GP in 2009) can be seen. Doubt about a fracture (followed by a re-examination after two days) existed for all of group 3 and part of group 2 in 2006, but only for part of group 3 in 2009. Similarly, clear differences in the treatment of fractures by the GP under the supervision of a surgeon can be seen zero in 2006; 69 in 2009. Moreover, all of the fractures for group 3 and most of those for group 2 could be treated by the GP under the guidance of a surgeon in 2009 (highlighted in red).

## Discussion

### Summary

There is a clear difference in outcomes between 2006 and 2009. Fewer fractures were missed and no severe fractures whatsoever were missed. Fewer patients had to make the unnecessary trip to the hospital five hours away. In 2006, 41 patients (13.1%) were found at the hospital to not have a fracture. In 2009, only 2 patients (0.4%) with a CT-scan indication were found to not have a fracture and therefore had travelled unnecessarily to the hospital. These differences ran parallel with the introduction of teleradiology into the general practice and yielded significant benefits for patients.

A further benefit of this introduction was that more fractures and dislocations could be treated by the GP. In 2009, 77 of the patients with fractures or dislocations (66.4%) could be treated by the GP under the supervision of a surgeon. In 2006, no patients could be treated in this manner. Moreover, in 2006 15 patients (22.5%) with mostly phalanx fractures or dislocations were treated by the general practitioner without x-ray confirmation of the fractures or dislocation, which is *not* in accordance with Dutch guidelines which require an x-ray confirmation [5,6].

An unexpected advantage of teleradiology was that immobilised patients with re-fractures as a result of new traumas could be diagnosed and treated by the GP. In previous years, this would have required a trip to the hospital.

An unexpected side effect was that more x-rays were taken with the availability of teleradiology, particularly for patients with uncertain clinical signs of fracture (patients in groups 2 and 3). Following the introduction of teleradiology, the percentage of patients with an unclear fracture returned for re-examination became more than twice as much than before. This suggests that the introduction of teleradiology created demand. However the introduction of teleradiology enables general practitioners to work in keeping with Dutch guidelines [5,6] and saves patients time, money and the anxiety of not knowing the outcome of a traumatic event.

The number of patients that visited the general practice with recent trauma is higher in 2009 compared to 2006. This increase can be partly explained by different weather conditions in 2009 which probably caused more risky outdoor activities as evidenced by the number of more severe (radius-ulna) fractures in 2009. In addition we cannot rule out that patients who previously went directly to the hospital prefer to visit the general practice after the introduction of teleradiology.

### Study strengths and limitations

Our study is the first to examine accurately diagnosed fractures, unnecessary trips to the hospital, treatment and number of x-rays before and after the introduction of teleradiology in a general practice. Information was obtained on initial and final diagnosis, subsequent treatment and number of x-rays made. The detailed description of the clinical signs and outcomes for trauma patients consulting a general practice before and after the introduction of teleradiology is thus a major strength of the present study. An additional strength is that the observed changes in the outcomes did not arise from differences in the x-ray examination procedures because, as usual in the Netherlands, all x-rays were interpreted by trained radiologists — both before and after the introduction of teleradiology.

A limitation is that we did not carry out a (randomized) controlled experiment, because of medical ethical reasons. Hence the difference in outcomes can in theory not only be attribute to the introduction of teleradiology. However since the GP’s, radiologists, surgeons, physical assistants and procedures were the same in 2006 and 2009, we have strong reasons to believe that the documented changes in outcomes are due to the introduction of teleradiology in the general practice.

Another possible limitation is that the samples from 2006 and 2009 were obviously obtained from different populations. Given that we could not contact all of the patients for follow up, there may have been more missed fractures. The number of research years (i.e., 2006 versus 2009) is small and also a possible limitation on the present study. Due to changes in the staffs of the general practice and the radiologists of the hospital radiology department in January 2010, it was not possible to continue data collection beyond 2009.

### Comparison to existing literature

Research on the introduction of teleradiology into primary healthcare is scarce and typically confined to the *implementation* of teleradiology and its costs, organization, logistics, management and disadvantages [1,3,7-11]. Benefits have also been described but are mainly based on the opinion of doctors and patients [9,12]. Studies of other telemedicine applications exist, including studies of the effects of telecardiology, videoconferencing and teledermatology [7,11-14]. The results of these studies are not sufficient, however, to justify the more widespread introduction of teleradiology into primary healthcare [7,11].

### Implications for future research and clinical practice

In a companion study, we performed a cost-benefit analysis of teleradiology and found its introduction into a general practice to result in a considerable reduction of costs not only for patients (111,000 euros per year) but also for health insurance companies (at least 89,000 euros per year) [15].

Future research should aim to implement teleradiology into more general practices and investigate whether the current procedure of having a trained radiologist interpret the x-rays can be expanded to allow GPs to be trained to also interpret x-rays. In addition, future research should certainly investigate the quality of the treatment by a GP, using teleradiology under the supervision of a surgeon, relative to the quality of the treatment by a surgeon in hospital.

## Conclusion

The present paper study suggests that teleradiology in a general practice has clear benefits in terms of reducing the number of missed fractures, unnecessary trips to the hospital and increasing the possibilities for treatment at home. Teleradiology is thus a good example of healthcare which can be transferred from hospitals to primary healthcare centres, despite the finding that following the Dutch Guidelines more x-rays were requested — particularly for patients with uncertain clinical signs of fractures. This conclusion presumably holds for other general practices in rural areas and other countries as well.

## References

[1] Mun SK, Tohne WG, Platenberg RC, Choi I (2005). Teleradiology and emerging business models. J Telemed Telecare.

[2] Knop FK, Staunting JA (2006). The benefits of diagnostic imaging in general practice. Ugeskr Laeger.

[3] Ekeland AG, Bowes A, Flottorp S (2010). Effectiveness of telemedicine: a systematic review of reviews. Int J Med Inform.

[4] EH L v, WJHM B v, Lagro-Janssen ALM, Schers HJ (2008). Ziekten in de Huisartsenpraktijk [Illnesses in General Practice]. Reed Business, fifth revised version.

[5] Belo J, Buis P, van Rijn R, Sentrop-Snijders E, Steenhuisen S, Wilkens C (2012). NHG-standaard Enkelbandletsels [NHG-guidelines Ankle Injuries]. Huisarts Wet.

[6] Keeman JN, Schadé E (1997). Spoedeisende Geneeskunde voor de Huisarts [Emergency Medicine for the GP. Bohn Stafleu Van Loghum, second revised version.

[7] Norum J, Pedersen S, Stømer J, Rumpsfeld M, Storma A, Jamissen N (2007). Priorisation of telemedicine services for large scale implementation in Norway. J Telemed Telecare.

[8] Char A, Kalyanpur A, Puttanna Gowda VN, Bharathi A, Singh J (2010). Teleradiology in an inaccessible area of northern India. J Telemed Telecare.

[9] Johansen I, Breivik E (2004). [English translation of title: is teleradiology service in primary health care cost-effective?]. Tisskr Nor Laegebforen.

[10] Krestin GP, Pieterman H (2011). Teleradiologie: bedreigingen en kansen [Teleradiology: threats and opportunities]. Ned Tijdschr Geneeskd.

[11] Hailey D, Roine R, Ohinmaa A (2002). Systematic review of evidence for the benefits of telemedicine. J Telemed Telecare.

[12] Moffat JJ, Eley DS (2010). The reported benefits of telehealth for rural Australians. Aust Health Rev.

[13] van der Heijden JP, de Keizer NF, Bos JD, Spuls PI, Witkamp L (2011). Teledermatology applied following patient selection by general practitioner in daily practice improves efficiency and quality of care at lower costs. Br J Dermatol.

[14] McGill AF, North JB (2012). Teleconference fracture clinics: a trial for rural hospitals. ANZ J Surg.

[15] Jacobs JJWM, Jacobs JPAM, Wiersma D, Sanderman R (2012). Teleradiologie in de huisartenspraktijk op Ameland. Een kosten-baten analyse [Teleradiology in general practice at Ameland. A cost-benefit analysis]. Ned Tijdschr Geneeskd.

